# Spatio-Temporal Variations in Malaria Incidence in Children Less than 10 Years Old, Health District of Sokone, Senegal, 2010–2013

**DOI:** 10.1371/journal.pone.0137737

**Published:** 2015-09-18

**Authors:** Emmanuelle Espié, Fatoumata Diene Sarr, Fodé Diop, Joseph Faye, Vincent Richard, Adama Tall, Aissatou Touré Baldé

**Affiliations:** 1 Epidemiology Unit, Pasteur Institute of Dakar, Dakar, Senegal; 2 Immunology Unit, Pasteur Institute of Dakar, Dakar, Senegal; 3 Laboratory of Parasitology-Biology, Faculty of Sciences and Technology, University Cheikh Anta Diop, Dakar, Senegal; Johns Hopkins Bloomberg School of Public Health, UNITED STATES

## Abstract

**Introduction:**

Malaria is a leading cause of morbidity and mortality in sub-Saharan Africa. Detailed characterization of the risks for malaria, among populations living in areas where the disease is endemic, is an important priority, especially for planning and evaluating future malaria-control tools. A prospective cohort study was implemented in children under ten years living in rural areas with high *Plasmodium falciparum* transmission in Senegal.

**Methods:**

Malaria incidence was prospectively evaluated over three year follow-up among a cohort of children aged less than 10 years old living in eight villages of the Sokone health district. The parents of 1316 children comprising a passive case detection cohort were encouraged to seek care from the study health centers at any time their child felt sick. In the event of reported history of fever within 24 hours or measured axillary temperature ≥ 37.5°C, a Rapid Diagnostic Test (RDT) was performed.

**Results:**

From November 2010 to October 2013, among the 1468 reported febrile episodes, 264 were confirmed malaria episodes. Over the 3 years, 218 (16.9%) children experienced at least one clinical malaria episode. Cumulative malaria incidence was 7.3 episodes per 100 children-year at risk, with remarkably heterogeneous rates from 2.5 to 10.5 episodes per 100 children-year at risk. Clinical malaria prevalence ranged from 11.5 to 28.4% in the high transmission season versus from 9.6 to 21.2% in the low transmission season.

**Conclusion:**

This longitudinal community-based study shows that occurrence of clinical malaria was not evenly distributed among all the cohort children in the eight villages. It demonstrates the complexity of spatial distribution of malaria incidence at a local level, even in a region of vegetation and altitudinal homogeneity.

## Introduction

Malaria is a leading cause of morbidity and mortality, particularly in children under five years living in sub-Saharan Africa, the infection being due to *Plasmodium falciparum* in 90% of cases [1]. Over the last five years, considerable efforts have been made to control malaria, leading to the decline in malaria transmission [1]. These changes are as the result of different intervention such as extensive use of insecticide-treated bednets and indoor residual spraying, improved malaria diagnosis with rapid diagnostic tests and effective Artemisin-based combination therapies. However, despite these significant progresses, control efforts remain inadequate, and malaria persists as a huge burden for many developing countries. To be most effective, the limited resources available for malaria control should be targeted at the populations in which they will have the greatest impact [2]. Detailed characterization of the risks for malaria, among populations living in areas where the disease is endemic, is an important priority, especially for planning and evaluating future malaria-control tools [3, 4]. Moreover, development and evaluation of malaria vaccine trials requests knowledge of the regional epidemiology of malaria [5]. Current malaria epidemiology data in endemic regions are essential to understand the relationships between transmission, immunity and malaria-related morbidity and mortality. Indeed, well-defined cohorts from the Gambia, Kenya and Tanzania have been instrumental in characterizing the epidemiology of malaria, exploring the acquisition of immunity, and demonstrating the effectiveness of control interventions such as vaccines [6–8].

If some established African institutions in the Gambia, Burkina Faso, Mali and Nigeria have already conducted clinical trials for candidate malaria vaccines, there is a need to strengthen and expand clinical trials sites in others West-African countries in anticipation of testing vaccine candidates that are currently in pre-clinical development. To address these challenges, the West African Network of Excellence for TB, AIDS and Malaria (WANETAM) has been created through the funding of the European and Developing Countries Clinical Trials Partnership (EDCTP), a European Union-funded and peer-review grant awarding agency [9]. In this context, in order to develop a Senegalese site in which an in-depth and comprehensive understanding of the epidemiology of malaria will be available, we conducted a 3 year-prospective study among children under ten years living in a rural area of Senegal. A multidisciplinary approach allowed for the simultaneous collection of a wide variety of epidemiologic, entomologic, and immunologic data from the same population over time.

This paper describes some of the fundamental parameters of malaria infection and disease, obtained by using passive case detection of malaria episodes, as it was experienced by the resident population during the study.

## Methods

### Study area and target population

The study was initiated in August 2010 in the health district of Sokone, region of Fatick. The region of Fatick is located in the Central-Southern part of Senegal, 280 km from Dakar, the capital. One reference district hospital and 16 peripheral health facilities constitute the government health network in this district. The climate is Sahelian, characterized by a rainy season, with 400–1000 mm of rain fall, from June to October and dry season from November to May.

**Fig 1:**
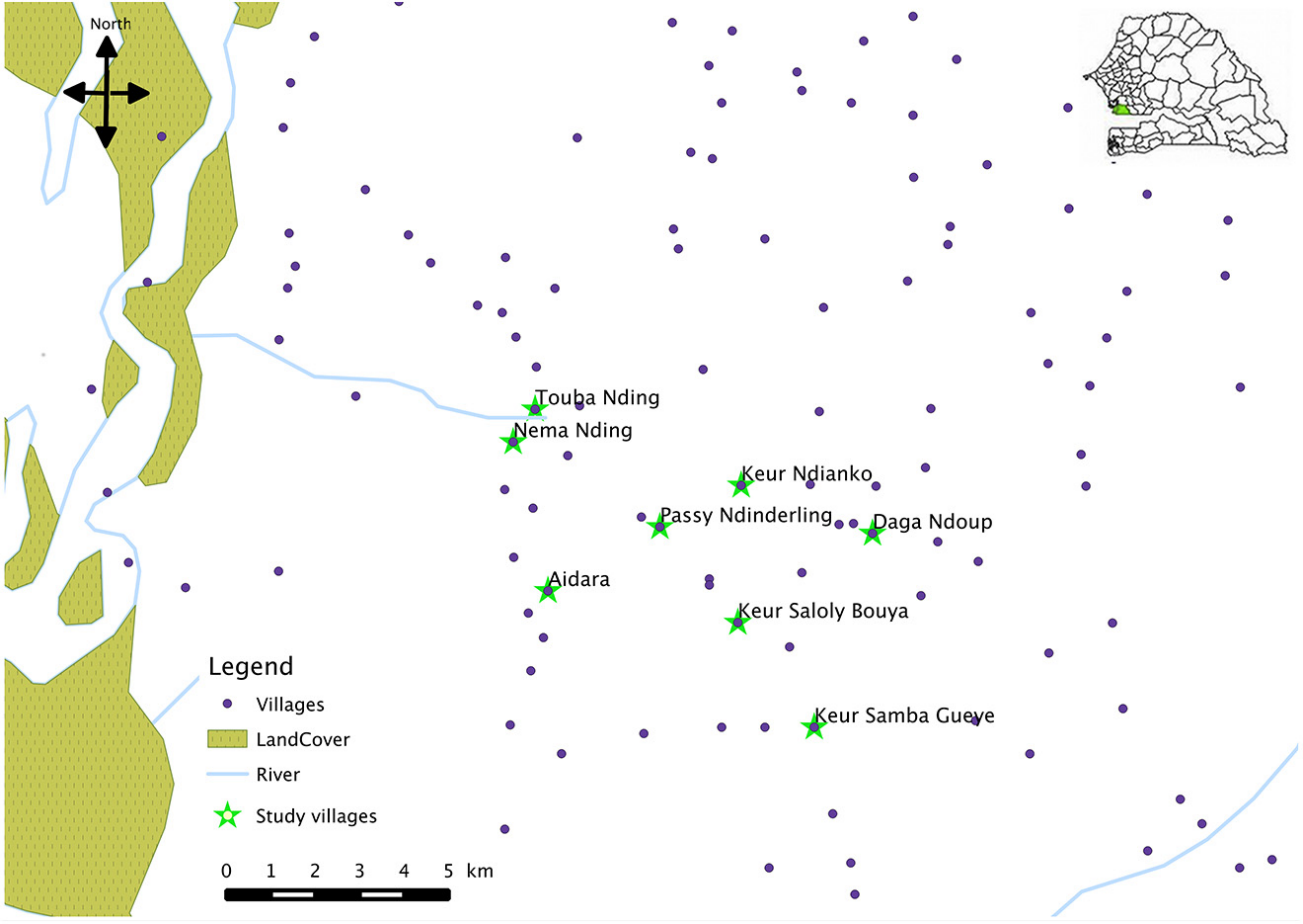
Map with the 8 villages involved in the study.

The study area included 8 villages and covers an area of 30 km^2^ (Fig 1). All 8 villages were situated within <6.5km of the two main peripheral health care centres, facilitating access to health care. The total resident population in the catchment area is estimated as 8000 inhabitants and culturally diverse with seven ethnic groups. The predominating ethnic group in the area is Wolof. Children below five years represent 19.4% of the population (around 1500). Most of the population depends on subsistence farming of cash crops (maize, millet, groundnuts and vegetables), and rearing of domestic animals (cattle, sheep, goat, chickens). Houses are typically made of mud walls and thatched or corrugated iron roofs.

Malaria transmission in the study area is markedly seasonal and intense during the rainy season (from June to October). The entomological inoculation rate varied from 4 to 30 infective bites/person/month during the rainy season of 2011 [10]. In the studied region, in 2008–2009, ITN coverage, measured by ownership of at least one mosquito net per household, reached 89% and approximately 52% of the population in general, and 65% of children <5 years, had slept under an Insecticide Treated Net the previous night [11]. In 2006, Artemisin-based combination therapies (ACTs) were introduced as first line treatment for uncomplicated childhood malaria and they were made freely available in 2010 [12].

### Census of the target population

In a first step, we conducted a census survey in order to identify the children who were born between the 1^st^ of October 2000 and the 30^th^ of September 2010 and who lived in one of the eight selected villages. During this census, a mapping of the villages was conducted with the recording of the longitude and latitude coordinates of the geographical center of the village and the identification of each house by a unique identification number.

After the head of households accepted to participate and sign the consent form, each child was provided with an identity study card with an unique identification number. The parents (or guardians or caretakers) were given instructions to go to the health centre in the event of any illness for free care.

### Inclusion for the cohort

The inclusion criteria were the following: children younger than 10 years, living in the study area, who were present at the beginning of the inclusion (1^st^ of November 2010) and had a blood sample.

At inclusion, a clinical examination of each child was done by a medical doctor and a blood sample was collected for identification of the blood group and type.

Among this cohort, a randomized sample of 330 children was selected using the full list of the included children by standardizing on the population size of the villages (+/- 10% of refusal and absence). Based on the percent of children 6–59 months of age with parasitemia due to *P*. *falciparum* found in the Fatick region in 2008 [11], the sample size was calculated in order to detect a prevalence of positive asexual parasitemia of 8% with a precision of 3% (risk of error = 5%).

On this sample, other blood samples were collected for complete blood count, and for preparation of duplicate thick blood smears. For children aged from 6 to 59 months old, anthropometrics measures (weight, height, mid-upper arm circumference) were also done by a nurse.

### Clinical follow-up during 3 years

From the 1^st^ of November 2010 to the 31^st^ of October 2013, a passive surveillance was undertaken and participants were encouraged to seek care at one of the two health centres within the study area for any illness. In these health centres providing routine care, local health staffs received training in study procedures and were provided standardized study form to record clinical information of children enrolled in the cohort.

In the event of reported history of fever within 24 hours or measured axillary temperature ≥ 37.5°C, a Rapid Diagnostic Test (RDT) was done for malaria confirmation.

Free drug treatment was available as indicated for malaria and non-malaria diseases. Cases of uncomplicated malaria were treated with a single weight-adjusted dose of Artemether-Lumefantrine or Artesunate-Amodiaquine, whereas cases of severe malaria were treated with parenteral quinine according to the Senegalese National Malaria programme recommendations.

During the three years, death was recorded and the cause of death reported by the parents was determinated using verbal autopsy.

### Statistical analysis

Estimation of mean, proportion and 95% confidence interval were calculated, using STATA software version 10 (StataCorp).

Prevalence of acute malnutrition was estimated on the basis of weight-for-height indices expressed in z-score. Anthropometric indices were calculated using Nutrisurvey software version 2007 (SMART, ENA) and compared against an international reference population using the 2005 WHO standards.

A clinical malaria episode was defined as an axillary temperature ≥ 37.5° or history of fever in last 24h and positive RDT.

Overall and village-specific malaria incidence rates over one year follow-up were calculated as the number of events malaria cases divided by the total person time at risk. Total person-time was calculated from date of enrolment to date of the departure outside the village.

Overall and village-specific malaria prevalence were calculated as the number of clinical malaria cases divided by the total of febrile cases reported during the same period.

### Ethical considerations

The study protocol and the informed consent form were approved by the National Ethical Committee for Research of Senegal. The study was conducted in compliance with principles set out by the Declaration of Helsinki, and the regulatory requirements of the Senegalese government on Health Research Ethic. Individual written informed consent was obtained from all children’s parents or legal representatives, in the presence of an independent witness for illiterate parents/legal representative.

## Results

### Census

During the census period (August-September 2010), a total of 396 households were enumerated in the eight selected villages. Among them, only 358 households had children younger than 10 years at the time of the census survey, representing a population of around 1800 children younger than 10 years; 62 families (17.3%) refused to participate to the study for cultural reasons. Finally, among the 296 families who accepted to participate, 1442 children younger than 10 years (720 girls and 722 boys) were identified. The age at the census varied from 10 days to 10 years (median 5 years) and 16% had less than 1 year old. The predominating ethnic group was Wolof (66%) following by Serer (16%) and Poular (9%).

### Baseline characteristics of the study cohort

Among the 1442 children, 1316 (91.3%) were finally included in the original cohort (Table 1). Among the 126 excluded, 4 died before inclusion, 47 were not present at the inclusion period and for 75, parents refused the blood sample. More than 90% of the total identified children were included.

**Table 1:** Distribution of village and individual characteristics among the 8 study villages, November 2010.

	Aidara	Daga Ndoup	Keur Ndianko	Keur Saloly Bouya	Keur Samba Guéye^b^	Nema Nding^b^	Passy Ndinderling	Touba Nding
**Total population** ^**a**^ **, N**	455	1215	811	1013	1824	1365	811	190
**Eligible children, n**	86	124	169	208	369	212	238	36
**Included children, n**	58 (67.4)	121 (97.6)	139 (80.3)	203 (97.6)	353 (95.7)	188 (88.7)	223 (93.7)	31 (86.1)
**Gender**								
** M**	28 (48)	61 (50)	66 (47)	105 (52)	175 (50)	92 (49)	109 (49)	19 (61)
** F**	30 (52)	60 (50)	73 (53)	98 (48)	178 (50	96 (51)	114 (51)	12 (39)
**Ethnic groups**								
** Wolof**	4 (7)	111 (92)	112 (81)	191 (94)	260 (74)	8 (4)	206 (92)	2 (6)
** Serer**	13 (22)	3 (2)	0	0	47 (13)	119 (63)	0	18 (58)
** Poular**	2 (3)	0	27 (19)	12 (6)	31 (9)	13 (7)	6 (3)	8 (26)
** Mandingue**	38 (66)	6 (5)	0	0	5 (1)	33 (18)	11 (5)	3 (10)
** Other**	1 (2)	1 (1)	0	0	10 (3)	15 (8)	0	0
**Birth season**								
** Dry**	52 (90)	104 (86)	122 (88)	167 (82)	293 (83)	141 (75)	177 (79)	25 (81)
** Rainy**	6 (10)	17 (14)	17 (12)	36 (18)	60 (17)	47 (25)	46 (21)	6 (19)
**Blood group**								
** O**	22 (38.6)	54 (45.0)	70 (52.6)	83 (42.1)	178 (51.0)	81 (43.1)	104 (47.5)	10 (32.3)
** A**	14 (24.6)	36 (30.0)	38 (28.6)	49 (24.9)	86 (24.6)	56 (29.8)	53 (24.3)	19 (61.3)
** B**	17 (29.8)	23 (19.2)	23 (17.3)	57 (28.9)	73 (20.9)	48 (25.5)	49 (22.4)	2 (6.5)
** AB**	4 (7.0)	7 (5.8)	2 (1.5)	8 (4.1)	12 (3.4)	3 (1.6)	13 (5.9)	0

Data are absolute number n (%) of children included in the analysis, unless otherwise indicated

a) based on population census data [year 2012]

b) villages where the health care centres are located

Among the 1316 included children in the cohort, 661 were female (sex-ratio M/F = 0.99). At inclusion, the median age was 5 years (range = 2 months-10 years), and 14% were less than 1 year old. The predominating ethnic group was Wolof (68%) following by Serer (15%) and Poular (8%) (Table 1). The socio-demographic characteristics of the cohort were not significantly different from those of the target population.

Among the 1307 children who had a determination of blood group and type, 609 (47%) were of O group and 1229 (954%) had a positive Rhesus factor. The predominant blood group and type was O+ (n = 567, 43.4%) following by A+ (n = 338, 25.9%) (Table 1).

Among the representative sample of 325 children, clinical examinations showed that 62 (19.1%) had fever (95%CI 15.0–23.9), one had splenomegaly (0.3%, 95%CI 0.02–2.0), and none had hepatomegaly. Among the 167 children aged between 6 and 59 months, 22.3% (95%CI 15.8–28.8) had acute malnutrition, and 4.5% (95%CI 1.2–7.7) severe acute malnutrition, according to 2005 WHO references.

Among the 293 children who had a complete blood count, 12 (4.1%; 95%CI, 2.2–7.2) had anemia (hemoglobin ≤ 7 g/dL), and one had severe anemia (hemoglobin ≤ 5g/dL).

Thick smears were done in 322 children during the rainy season. Among them, 303 were negative, 3 had gametocytes of *Plasmodium falciparum* and 16 had trophozoites of *Plasmodium falciparum* (Table 2). No thick smear was positive for *P*. *ovale*, *P*. *malariae* or Borrelia spp. Among the 16 children who had a positive smear, 5 had clinical symptoms of malaria at the time or within the two following days of the blood sample. Prevalence of parasitemia (asexual *P falciparum* infection) was 3.4% (95%CI, 1.8–6.2). Geometric mean parasite density was 12 459 parasites/μL (95%CI, 0–33 620).

**Table 2:** Malaria prevalence and malariometric indices among the randomly selected sample of 325 children, November 2010.

Clinical malaria	*P falciparum* infection	Splenomegaly	Hemoglobin	Packed cell volume
n (%)	n (%)	n (%)	mean (IQR)	mean (IQR)
5 (1.6)	16 (5.0)	1 (0.3)	10.5 (9.6–11.5)	32.3 (30.2–35.0)

IQR = interquartile range

In three villages (Daga Ndoup, Keur Ndianko and Touba Nding), no asymptomatic carriage was detected; all the thick smears tested were negative.

### Clinical follow-up and malaria incidence rates

From 1^st^ of November 2010 to 31^st^ of October 2013, 3264 visits for clinical episodes were notified by the two health centres’ nurses: 1524 during the first year, 720 during the second year and 1020 during the last year.

Among the 1202 children who have been follow-up during the 3 years, 188 (15.6%) children had no clinical episode, notified by the health centre. Among the children who had at least one visit per year, between 14.3% and 35.6% had more than one by year. Annual number of episodes per child ranged from 1 to 9.

**Fig 2:**
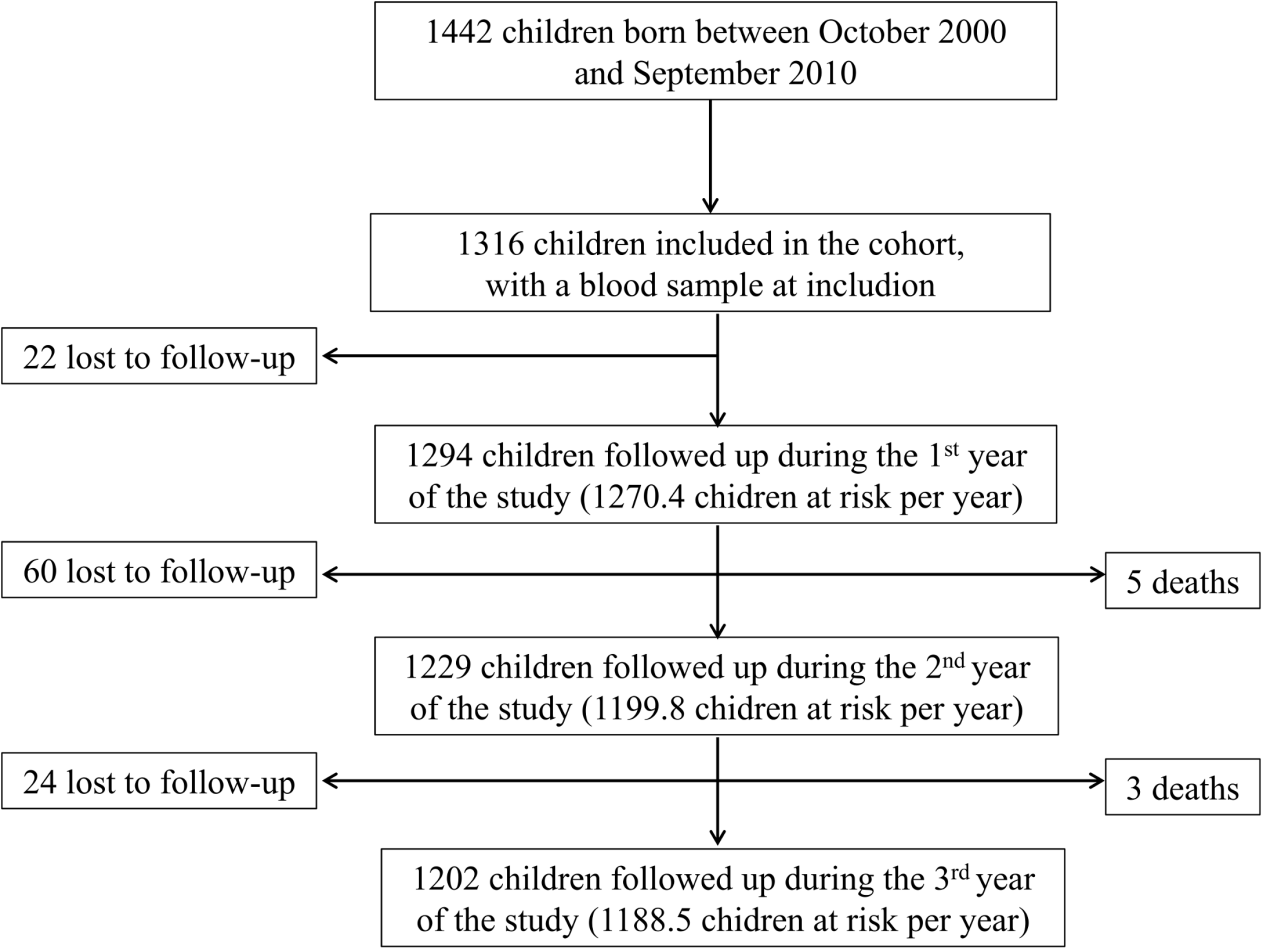
Flow chart of the follow-up of the cohort.

During the 3 years-follow-up period (Fig 2), 84 (6.4%) children left the study area and 8 died. Five deaths were reported in 2011 and 3, in 2012. The causes of death reported by the parents were severe malaria (n = 4, 62.5%), snake bite, severe burn, severe respiratory infection and unknown. Losses to follow up were mainly due to parents moving away from the study area: to another region of Senegal (70%) or to Gambia (n = 21, 25%), or to the death of one of the parents (n = 4, 5%).

Among the 3264 visits for clinical episodes, 1463 (44.8%) were febrile clinical episode. The duration of the symptoms reported by the parents, before the visit to the health centre varied between 1 and 10 days with a median of 3 days (mean = 2.6 days). The main infections identified by the nurses were acute respiratory infections (n = 629, 43.0%), followed by confirmed clinical malaria (n = 274, 18.8%) (Table 3).

**Table 3:** Annual distribution of diagnosis reported by the heath centres’ nurses, November 2010-October 2013

Number (%)	Year 1	Year 2	Year 3
	2010–2011	2011–2012	2012–2013
Lower respiratory tract diseases	312 (42)	147 (51)	170 (39)
Clinical malaria confirmed by RDT	134 (18)	30 (11)	110 (26)
Enteric infections	71 (10)	24 (8)	18 (4)
Other reported diagnosis^a^	155 (21)	54 (19)	51 (12)
Unkown febrile diseases	24 (3)	10 (3)	76 (17)
Unknown diagnosis	40 (6)	21 (7)	10 (2)
**Total**	741 (100)	287 (100)	435 (100)

^*a*^ *Skin diseases*, *buccal infections*, *ear and ocular infections*, *etc*.

Across the 3 years of follow-up, annual clinical malaria prevalence varied widely (Table 3). Clinical malaria prevalence ranged from 11.5 to 28.4% in the high transmission season versus from 9.6 to 21.2% in the low transmission season. The rates of malaria disease in the successive years and seasons of follow up were significantly different from each other (p-value<0.03).

The overall malaria incidence was 7.3 (95% confidence interval [95% CI], 6.5–8.3) episodes per 100 children-year at risk. However, malarial incidence was remarkably heterogeneous, resulting in a range of incidence rates from 2.5 to 10.5 episodes per 100 children-year at risk (Table 4). The rates of disease in the successive years of follow up were statistically significantly different from each other.

**Table 4:** Year-specific incidence of confirmed clinical malaria episodes.

Year of follow-up	Number of children	Number of fever episodes	Number of malaria	Total children-years at risk	Incidence rate (95%CI) per 100 ch-yr at risk
2010–2011	1294	741	134	1270.4	10.5 (8.6–12.0)
2011–2012	1229	287	30	1199.8	2.5 (1.6–3.4)
2012–2013	1202	433	110	1188.5	9.2 (7.5–11.1)

Of the 1316 children enrolled in the study, 1094 (83.1%) experienced no episodes of malaria over the 3 years. Among the children who experienced at least one episode during the entire follow-up, between 30 and 45% experienced one episode per year, and between 0.5 and 1.7% experienced more than 5 episodes per year (maximum, 9 episodes per year). Malaria incidence varied considerably by calendar period, ranging from 1.2 episodes per 100 children-year (November 2011-May 2012) to 7.6 episodes per 100 children-year (November 2010-May 2011), with peaks corresponding with the periods during and after heavy rains.

**Fig 3:**
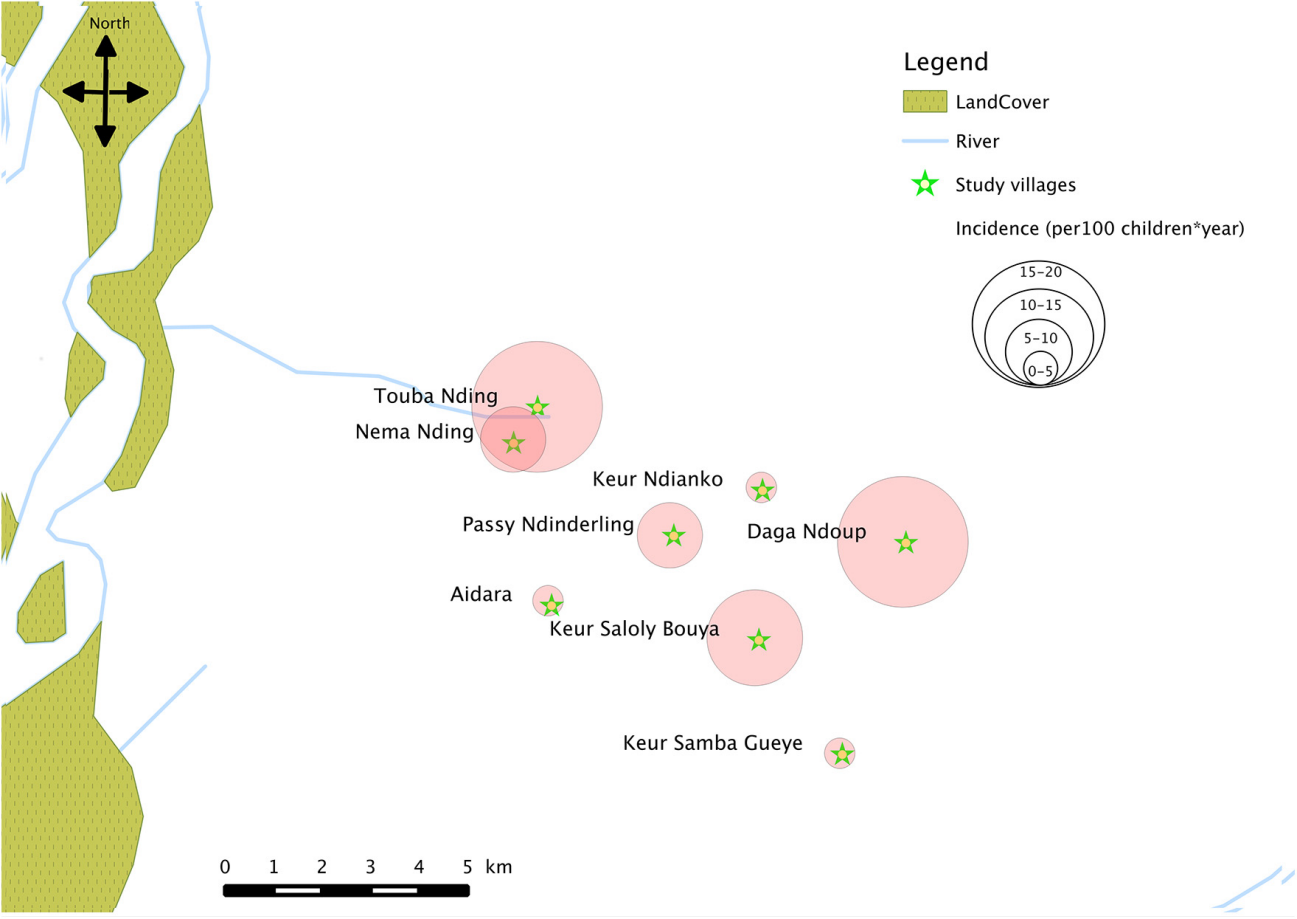
Spatial distribution of overall incidence of confirmed clinical malaria episodes in the study area.

Incidence rates differed greatly between the 8 villages, with the lowest being 1.1 (95% CI, 0.3–2.9) episodes per 100 children-year in Keur Ndianko and the highest being 17.8 (95% CI, 10.2–28.9) episodes per 100 children-year in Touba Nding for the entire observation time (Table 5, Fig 3).

**Table 5:** Year-specific incidence of confirmed clinical malaria episodes by village.

Village	Coordinates	Incidence	rate (95%CI)	per 100 ch-yr	at risk
	Longitude Latitude	2010–2011	2011–2012	2012–2013	Overall
**Aidara**	16° 25' 0.1"W	5.6	1.9	0	2.5
	13° 40' 59.9"N	(1.2–15.7)	(0.5–10.4)	(0–7.2)	(0.7–6.7)
Daga Ndoup	16° 21' 45.0"W	31.4	2.6	14.3	16.1
	13° 40' 78.2"N	(23.1–40.5)	(0.5–7.3)	(8.5–21.9)	(11.9–20.5)
Keur Ndianko	16° 22' 50.7"W	0.8	0	2.6	1.1
	13° 42' 28.5"N	(0.2–4.3)	(0–3.1)	(0.5–7.4)	(0.3–2.9)
Nema Nding	16° 25' 57.5"W	21.4	0.6	2.8	8.4
	13° 42' 83.1"N	(15.7–28.0)	(0.1–3.1)	(0.9–6.4)	(6.2–11.3)
Passy Ndinderling	16° 23' 69.2"W	5.6	4.5	13.8	7.9
	13° 41' 85.5"N	(2.9–9.5)	(2.0–8.3)	(9.4–19.4)	(5.8–10.4)
Keur Saloly Bouya	16° 21' 62.4"W	5.7	4.4	21.2	10.1
	13° 40' 54.6"N	(2.9–9.9)	(1.9–8.4)	(15.5–27.9)	(7.6–13.1)
**Keur Samba Guéye**	16° 21' 28.8"W	3.8	1.9	5.1	3.6
	13° 39' 21.1"N	(2.0–6.4)	(0.7–4.0)	(2.9–8.1)	(2.5–4.9)
Touba Nding	16° 25' 24.4"W	41.9	3.4	6.7	17.8
	13° 43' 32.9"N	(24.5–60.9)	(0.8–17.2)	(0.8–22.1)	(10.2–28.9)

## Discussion

This longitudinal community-based study carried out in a rural area of Senegal with 3 years of follow-up confirms the findings of other studies that have shown spatio-temporal clustering of malaria cases at varying geographical extents [13–16].

The results of the purely temporal analysis of malaria data by year showed that prevalence and incidence rate varied significantly from one year to another, with a decrease between 2011 and 2012 followed by an increase between 2012 and 2013. In Senegal, since 2010, the trend in the number of malaria cases decreased with a lowest number reported in 2012 [1]. These trends are also observed in a well-studied cohort in the villages of Ndiop and Dielmo, located in the same area as our study villages [17, 18]. As the meteorological parameters have close links with the variation of malaria incidence, climate impacts on the malaria transmission has to be explored to explain the significant reduction or increase of malaria cases.

Purely spatial analysis shows that occurrence of clinical malaria was not evenly distributed among all the cohort children in the eight villages. It demonstrates the complexity of spatial distribution of malaria incidence at a local level, even in a region of vegetation and altitudinal homogeneity. Thus varying malaria attack rates were observed in the villages of our study area, which were at most 5 km distant from each other. These results was confirmed by those obtained from an entomologic survey [10] and a bednets use survey implemented in March 2013 *[data not shown]*.Thus in villages with the higher malaria incidence, a high level of transmission was recorded based on a higher entomological inoculation rate (EIR) and a lower use of bednets. An 8-fold variation of EIR was observed between the village with the higher incidence of malaria and the village with the lower incidence, in terms of transmission potential [10]. In any case, the clusters of higher malaria incidence could not be explained by closer vicinity to health care facilities—that is, a higher likelihood that children whose households were located near the health care centre would report at the health centre with suspected malaria. Variation in the risk of malaria over short distances, between neighboring villages in endemic regions has long been recognized [19, 20]. At present, the factors underlying the microepidemiology of malaria are not fully understood, but include variation in distance to the nearest mosquito breeding site, water body or vegetation, household structural features [21, 22], and both human behavioural and genetic factors [22], that may also result in differential attractiveness to mosquitoes. Unfortunately during our study, these environmental factors (e.g. vegetation density, rivers, seasonally flooded bottomlands…) and other risk factors have not been explored over the three years of the study and no mapping of locations of breeding sites and children houses in the individual villages was performed.

In Africa, longitudinal studies in well-defined cohorts are critical to improving the understanding of the complex epidemiology of malaria, in rural villages where stable populations are well-suited to participate. Thus, even if our study provided valuable data on malaria epidemiology in the studied area, it has its own limitations. The passive cases detection may have conducted to underestimation, but it should have been quite limited as free drug treatment was available for malaria and non-malaria illnesses at the two health care centres, and parents of children were more likely to seek medical care for symptomatic illness as they were encouraged by field workers during annual visits. However, in one village (Aidara), the relationship between the families and the medical staff of one of the health care centre during the last year of the study have deteriorated to the point that families decided to stop to go to this health care centre. So the data reported for this village was totally biased for the third year of follow-up. In our study, malaria diagnosis was based on RDT without microscopic examination of thick and thin blood smears. This absence of *P*. *falcifarum* density evaluation, without taking in account threshold levels of parasitemia to assess malaria morbidity, might be induced an overestimation of incidence rates in this region [23].

Although measures of socio-economic status were not controlled, the fact that the bednets survey had been conducted at the end of the follow-up period and the well-know beneficit of bednet use in children through nets distribution campaigns, may overestimate the real use of bednets given the higher reported use of bednets and the lower quality and age of bednets noted when nets were directly observed in the households during the survey. Thus even if the use of bed nets was relatively common among all the villages, most of them were old, with holes and probably not more treated by insecticide. The loss of insecticidal protective effect could explain the increased risk of mosquito bites and the increase in malaria incidence during the third year of the clinical follow-up.

In summary, even in small geographic areas, malaria transmission shows heterogeneity. The epidemiology of malaria in the study area may be unique, and this will limit the generalizability of the findings, but has several implications. First, the study of malaria in such a micro-environment may help to provide better knowledge on how the disease transmission is influenced by preventive and control measures. Second, taking in account variability in malaria risk in small areas might be important for the planning and conduct of interventional trials and appropriate analyses should be applied to overcome the unbalanced distribution of risk factors and confounders in apparently homogeneous areas.

## References

[1] WHO Global Malaria Programme: World Malaria Report 2013. Geneva; 2013.

[2] Woolhouse ME, Dye C, Etard JF, Smith T, Charlwood JD, Garnett GP et al Heterogeneities in the transmission of infectious agents: implications for the design of control programs. Proc Natl Acad Sci USA 1997; 94:338–342. 8990210 10.1073/pnas.94.1.338 PMC19338

[3] Bousema T, Griffin JT, Sauerwein RW, Smith DL, Churcher TS, Takken W, et al Hitting hotspots: spatial targeting of malaria for control and elimination. PLoS Med 2012; 9(1):e1001165 10.1371/journal.pmed.1001165 22303287 PMC3269430

[4] Carter R, Mendis KN, Roberts D. Spatial targeting of interventions against malaria. Bull World Health Organ 2000; 78(12):1401–1411. 11196487 PMC2560653

[5] Greenwood B. Malaria vaccines. Evaluation and implementation. Acta Trop 2005; 95(3):298–304 15955523 10.1016/j.actatropica.2005.04.017

[6] Alonso PL, Sacarlal J, Aponte JJ, Leach A, Macete E, Milman J, et al Efficacy of the RTS,S/AS02A vaccine against *Plasmodium falciparum* infection and disease in young African children: randomised controlled trial. Lancet 2004; 364(9443):1411–1420. 15488216 10.1016/S0140-6736(04)17223-1

[7] Bloland PB, Boriga DA, Ruebush TK, McCormick JB, Roberts JM, Oloo AJ et al Longitudinal cohort study of the epidemiology of malaria infections in an area of intense malaria transmission II. Descriptive epidemiology of malaria infection and disease among children. Am J Trop Med Hyg 1999; 60(4):641–648. 10348241 10.4269/ajtmh.1999.60.641

[8] Schellenberg D, Menendez C, Aponte JJ, Kahigwa E, Tanner M, Mshinda H, et al Intermittent preventive antimalarial treatment for Tanzanian infants: follow-up to age 2 years of a randomised, placebo-controlled trial. Lancet 2005; 365(9469):1481–1483. 15850632 10.1016/S0140-6736(05)66418-5

[9] Miiro GM, Oukem-Boyer OO, Sarr O, Rahmani M, Ntoumi F, Dheda K, et al NoEs' programme. EDCTP regional networks of excellence: initial merits for planned clinical trials in Africa. BMC Public Health 2013; 13:258 10.1186/1471-2458-13-258 23517572 PMC3623728

[10] Niang el HA, Touré A, Ngom el HM, Konaté L, Faye O, Diallo M, et al Malaria transmission pattern in an area selected for clinical trials in the sudanian area of senegal (West Africa). J Trop Med 2013; 2013: 907375 10.1155/2013/907375 23476671 PMC3576792

[11] Ndiaye S, Ayad M. Enquête Nationale sur le Paludisme au Sénégal 2008–2009 (ENPS II). Calverton, Maryland, USA: Centre de Recherche pour le Développement Humain [Sénégal] et ICF Macro 7 2009 Available: http://pdf.usaid.gov/pdf_docs/PNADQ631.pdf

[12] Giardina F, Gosoniu L, Konate L, Diouf MB, Perry R, Gaye O, et al Estimating the burden of malaria in Senegal: Bayesian zero-inflated binomial geostatistical modeling of the MIS 2008 data. PLoS One 2012; 7(3):e32625 10.1371/journal.pone.0032625 22403684 PMC3293829

[13] Wen L, Li C, Lin M, Yuan Z, Huo D, Li S, et al Spatio-temporal analysis of malaria incidence at the village level in a malaria-endemic area in Hainan, China. Malar J 2011; 10:88 10.1186/1475-2875-10-88 21492475 PMC3094226

[14] Wangdi K, Kaewkungwal J, Singhasivanon P, Silawan T, Lawpoolsri S, White NJ. Spatio-temporal patterns of malaria infection in Bhutan: a country embarking on malaria elimination. Malar J 2011; 10:89 10.1186/1475-2875-10-89 21496285 PMC3094227

[15] Yeshiwondim AK, Gopal S, Hailemariam AT, Dengela DO, Patel HP. Spatial analysis of malaria incidence at the village level in areas with unstable transmission in Ethiopia. Int J Health Geogr 2009; 8:5 10.1186/1476-072X-8-5 19171051 PMC2646707

[16] Mirghani SE, Nour BY, Bushra SM, Elhassan IM, Snow RW, Noor AM. The spatial-temporal clustering of *Plasmodium falciparum* infection over eleven years in Gezira State, The Sudan. Malar J 2010; 9:172 10.1186/1475-2875-9-172 20565854 PMC2903606

[17] Roucher C, Rogier C, Sokhna C, Tall A, Trape JF. A 20-Year longitudinal study of *Plasmodium ovale* and *Plasmodium malariae* prevalence and morbidity in a West African population. PLoS One 2014; 9(2):e87169 10.1371/journal.pone.0087169 24520325 PMC3919715

[18] Trape JF, Tall A, Sokhna C, Ly AB, Diagne N, Ndiath O, et al The rise and fall of malaria in a West African rural community, Dielmo, Senegal, from 1990 to 2012: a 22 year longitudinal study. Lancet Infect Dis 2014; 14(6):476–488. 10.1016/S1473-3099(14)70712-1 24813159

[19] Greenwood BM. The microepidemiology of malaria and its importance to malaria control. Trans R Soc Trop Med Hyg 1989; 83 Suppl:25–29. 2576161 10.1016/0035-9203(89)90599-3

[20] Lindsay SW, Campbell H, Adiamah JH, Greenwood AM, Bangali JE, Greenwood BM. Malaria in a peri-urban area of The Gambia. Ann Trop Med Parasitol 1990; 84:553–562. 2076033 10.1080/00034983.1990.11812510

[21] Bousema T, Drakeley C, Gesase S, Hashim R, Magesa S, Mosha F, et al Identification of hot spots of malaria transmission for targeted malaria control. J Infect Dis 2010; 201(11):1764–1774. 10.1086/652456 20415536

[22] Kreuels B, Kobbe R, Adjei S, Kreuzberg C, von Reden C, Bäter K, et al Spatial variation of malaria incidence in young children from a geographically homogeneous area with high endemicity. J Infect Dis 2008; 197(1):85–93. 10.1086/524066 18171290

[23] Roucher C, Rogier C, Dieye-Ba F, Sokhna C, Tall A, Trape JF. Changing malaria epidemiology and diagnostic criteria for *Plasmodium falciparum* clinical malaria. PLoS One 2012;7(9):e46188 10.1371/journal.pone.0046188 23029433 PMC3460864

